# Bis[2-(benzyl­imino­meth­yl)pyrrol-1-ido-κ^2^ *N*,*N*′]bis­(dimethyl­amido-κ*N*)titanium(IV)

**DOI:** 10.1107/S1600536812014365

**Published:** 2012-04-13

**Authors:** Zhou Chen, Yonglu Liu, Yahong Li, Bin Hu

**Affiliations:** a Qinghai Institute of Salt Lakes, Chinese Academy of Sciences, Xining 810008, People’s Republic of China; b Key Laboratory of Organic Synthesis of Jiangsu Province, College of Chemistry, Chemical Engineering and Materials Science, Soochow University, Suzhou 215123, People’s Republic of China

## Abstract

The mononuclear title complex, [Ti(C_2_H_6_N)_2_(C_12_H_11_N_2_)_2_], was synthesized by the reaction of 1-phenyl-*N*-[(pyrrol-2-yl)methyl­idene]methanamine with Ti(NMe_2_)_4_. The Ti^IV^ atom is coordinated in a distorted octa­hedral geometry by four N atoms from two derivatized methanamine ligands and two N atoms from two dimethyl­amide ions. The dihedral angles between the pyrrole and phenyl rings in the bidentate ligands are 62.36 (9) and 78.32 (8)°. In the crystal, a weak π–π stacking inter­action [centroid–centroid distance = 3.864 (2) Å] involving centrosymmetrically related mol­ecules is observed.

## Related literature

For the synthesis of *N*-[(pyrrol-2-yl)methyl­ene]-1-phenyl­methanamine, see: Brunner *et al.* (1998 ▶); Joly & Jacobsen (2004 ▶); La Regina *et al.* (2007 ▶). For the structures of related complexes, see: Li *et al.* (2008 ▶); Brunner *et al.* (2003 ▶); Simpson *et al.* (2004 ▶); Wansapura *et al.* (2003 ▶); Beer *et al.* (2003 ▶).
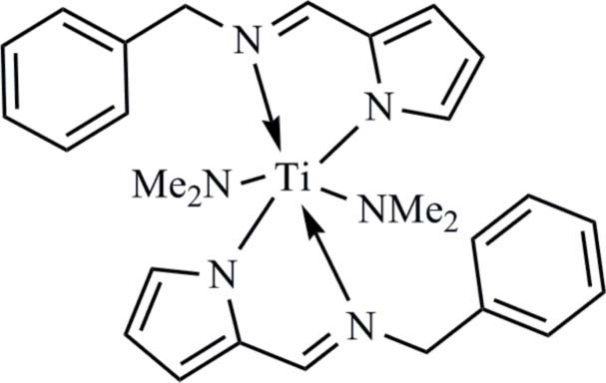


## Experimental

### 

#### Crystal data

[Ti(C_2_H_6_N)_2_(C_12_H_11_N_2_)_2_]
*M* *_r_* = 502.48
Triclinic, 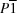

*a* = 8.6363 (17) Å
*b* = 9.887 (2) Å
*c* = 16.666 (3) Å
α = 77.15 (3)°
β = 84.72 (3)°
γ = 71.82 (3)°
*V* = 1317.8 (4) Å^3^
*Z* = 2
Mo *K*α radiation
μ = 0.35 mm^−1^
*T* = 293 K
0.25 × 0.23 × 0.20 mm

#### Data collection

Bruker APEXII CCD diffractometer
Absorption correction: multi-scan (*SADABS*; Bruker, 2005 ▶) *T*_min_ = 0.917, *T*_max_ = 0.933
6581 measured reflections
4578 independent reflections
3886 reflections with *I* > 2σ(*I*)
*R*_int_ = 0.024

#### Refinement

*R*[*F* ^2^ > 2σ(*F* ^2^)] = 0.039
*wR*(*F* ^2^) = 0.121
*S* = 1.08
4578 reflections
320 parameters
H-atom parameters constrained
Δρ_max_ = 0.32 e Å^−3^
Δρ_min_ = −0.33 e Å^−3^

### 

Data collection: *APEX2* (Bruker, 2005 ▶); cell refinement: *SAINT* (Bruker, 2005 ▶); data reduction: *SAINT*; program(s) used to solve structure: *SHELXS97* (Sheldrick, 2008 ▶); program(s) used to refine structure: *SHELXL97* (Sheldrick, 2008 ▶); molecular graphics: *SHELXTL* (Sheldrick, 2008 ▶); software used to prepare material for publication: *SHELXTL*.

## Supplementary Material

Crystal structure: contains datablock(s) I, global. DOI: 10.1107/S1600536812014365/rz2726sup1.cif

Structure factors: contains datablock(s) I. DOI: 10.1107/S1600536812014365/rz2726Isup2.hkl

Additional supplementary materials:  crystallographic information; 3D view; checkCIF report

## Figures and Tables

**Fig. 1.**
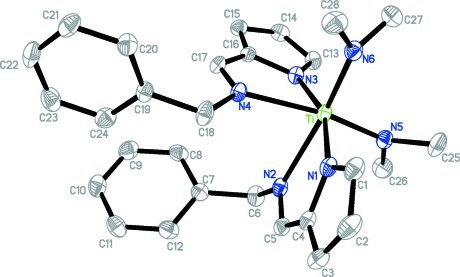
The molecular structure of the title compound, with 30% probability displacement ellipsoids. H atoms have been omitted for clarity.

## supplementary crystallographic information

### Comment

The ligand *N*-[(pyrrol-2-yl)methylene]-1-phenylmethanamine can be synthesized by different methods (Brunner *et al.*, 1998; Joly & Jacobsen, 2004; La Regina *et al.*, 2007). This ligand has been used in the synthesis of a series of metal-organic complexes such as Ir(III) (Li *et al.*, 2008), Rh(III) (Brunner *et al.*, 2003), Pd(II) (Simpson *et al.*, 2004), Cu(II) (Wansapura *et al.*, 2003), Zn(II) and Ni(II) (Beer *et al.*, 2003) by the reaction of the ligand with metal salts. Herein we report the synthesis and crystal structure of a titanium(IV) complex of this ligand.

The molecular structure of the mononuclear title complex is shown in Fig. 1. A distorted octahedral coordination geometry about the metal atom is provided by four nitrogen atoms from two *N*-[(pyrrol-2-yl)methylene]-1-phenylmethanamine ligands and two nitrogen atoms from two *cis*-arranged dimethylamino ions. In the bidentate ligands, the dihedral angles formed by the pyrrole and phenyl rings are 62.36 (9) and 78.32 (8)°. In the crystal structure, a weak π—π stacking interaction involving the C7–C12 phenyl rings of centrosymmetrically-related molecules with a centroid-to-centroid distance of 3.864 (2) Å is observed.

### Experimental

To a solution of Ti (NMe_2_)_4_ (0.112 g, 0.5 mmol) in THF (2 mL) was added *N*-((pyrrol-2-yl)methylene)-1 phenylmethanamine (0.184 g, 1 mmol) in THF (3 mL). After stirring at room temperature overnight, volatiles were removed *in vacuo*, resulting in an orange solid (0.236 g, yield 94 %). Single crystals suitable for X-ray diffraction were grown from a toluene/hexane (1:1 *v*/*v*) solution at -35°C.

### Refinement

All H atoms were positioned geometrically and refined using a riding model, with C—H = 0.93–0.97 Å and with *U*_iso_(H) = 1.2*U*_eq_(C) or 1.5*U*_eq_(C) for methyl H atoms.

### Crystal data

**Table:**

[Ti(C_2_H_6_N)_2_(C_12_H_11_N_2_)_2_]	*Z* = 2
*M**_r_* = 502.48	*F*(000) = 532
Triclinic, *P*1	char
Hall symbol: -P 1	*D*_x_ = 1.266 Mg m^−^^3^
*a* = 8.6363 (17) Å	Mo *K*α radiation, λ = 0.71073 Å
*b* = 9.887 (2) Å	Cell parameters from 5124 reflections
*c* = 16.666 (3) Å	θ = 2.4–27.7°
α = 77.15 (3)°	µ = 0.35 mm^−^^1^
β = 84.72 (3)°	*T* = 293 K
γ = 71.82 (3)°	Block, yellow
*V* = 1317.8 (4) Å^3^	0.25 × 0.23 × 0.20 mm

### Data collection

**Table:**

Bruker APEXII CCD diffractometer	4578 independent reflections
Radiation source: fine-focus sealed tube	3886 reflections with *I* > 2σ(*I*)
Graphite monochromator	*R*_int_ = 0.024
φ and ω scans	θ_max_ = 25.0°, θ_min_ = 2.2°
Absorption correction: multi-scan (*SADABS*; Bruker, 2005)	*h* = −10→9
*T*_min_ = 0.917, *T*_max_ = 0.933	*k* = −11→10
6581 measured reflections	*l* = −19→16

### Refinement

**Table:**

Refinement on *F*^2^	Primary atom site location: structure-invariant direct methods
Least-squares matrix: full	Secondary atom site location: difference Fourier map
*R*[*F*^2^ > 2σ(*F*^2^)] = 0.039	Hydrogen site location: inferred from neighbouring sites
*wR*(*F*^2^) = 0.121	H-atom parameters constrained
*S* = 1.08	*w* = 1/[σ^2^(*F*_o_^2^) + (0.0714*P*)^2^ + 0.1031*P*] where *P* = (*F*_o_^2^ + 2*F*_c_^2^)/3
4578 reflections	(Δ/σ)_max_ = 0.001
320 parameters	Δρ_max_ = 0.32 e Å^−^^3^
0 restraints	Δρ_min_ = −0.33 e Å^−^^3^

### Special details

**Table:**

Geometry. All e.s.d.'s (except the e.s.d. in the dihedral angle between two l.s. planes) are estimated using the full covariance matrix. The cell e.s.d.'s are taken into account individually in the estimation of e.s.d.'s in distances, angles and torsion angles; correlations between e.s.d.'s in cell parameters are only used when they are defined by crystal symmetry. An approximate (isotropic) treatment of cell e.s.d.'s is used for estimating e.s.d.'s involving l.s. planes.
Refinement. Refinement of *F*^2^ against ALL reflections. The weighted *R*-factor *wR* and goodness of fit *S* are based on *F*^2^, conventional *R*-factors *R* are based on *F*, with *F* set to zero for negative *F*^2^. The threshold expression of *F*^2^ > σ(*F*^2^) is used only for calculating *R*-factors(gt) *etc*. and is not relevant to the choice of reflections for refinement. *R*-factors based on *F*^2^ are statistically about twice as large as those based on *F*, and *R*- factors based on ALL data will be even larger.

### Fractional atomic coordinates and isotropic or equivalent isotropic displacement parameters (Å^2^)

**Table:**

	*x*	*y*	*z*	*U*_iso_*/*U*_eq_	
Ti	0.18544 (4)	0.26945 (3)	0.33887 (2)	0.02838 (14)	
N1	0.0595 (2)	0.16019 (17)	0.28584 (11)	0.0320 (4)	
N2	0.2912 (2)	0.27040 (17)	0.20770 (10)	0.0320 (4)	
N4	0.0277 (2)	0.48183 (17)	0.26901 (10)	0.0304 (4)	
N3	0.2968 (2)	0.42386 (17)	0.35233 (10)	0.0309 (4)	
N5	0.3662 (2)	0.10471 (18)	0.37812 (11)	0.0359 (4)	
N6	0.0465 (2)	0.27976 (18)	0.43391 (11)	0.0371 (4)	
C7	0.3511 (3)	0.4759 (2)	0.10416 (13)	0.0347 (5)	
C14	0.4206 (3)	0.5713 (2)	0.38831 (14)	0.0393 (5)	
H14	0.4952	0.6017	0.4110	0.047*	
C17	0.0711 (2)	0.5917 (2)	0.27540 (13)	0.0316 (5)	
H17	0.0121	0.6853	0.2503	0.038*	
C16	0.2107 (2)	0.5676 (2)	0.32156 (12)	0.0294 (4)	
C19	−0.1969 (2)	0.6496 (2)	0.17407 (13)	0.0323 (5)	
C4	0.1141 (3)	0.1328 (2)	0.20891 (13)	0.0330 (5)	
C5	0.2381 (3)	0.1951 (2)	0.17016 (13)	0.0342 (5)	
H5	0.2795	0.1808	0.1182	0.041*	
C13	0.4227 (3)	0.4287 (2)	0.39300 (13)	0.0362 (5)	
H13	0.5005	0.3473	0.4205	0.043*	
C1	−0.0532 (3)	0.0895 (2)	0.30949 (15)	0.0401 (5)	
H1	−0.1108	0.0885	0.3595	0.048*	
C20	−0.3424 (3)	0.7220 (2)	0.20766 (14)	0.0397 (5)	
H20	−0.3826	0.6768	0.2565	0.048*	
C15	0.2844 (3)	0.6603 (2)	0.34270 (13)	0.0359 (5)	
H15	0.2502	0.7614	0.3293	0.043*	
C6	0.4175 (3)	0.3353 (2)	0.16516 (14)	0.0409 (5)	
H6A	0.4937	0.2657	0.1365	0.049*	
H6B	0.4776	0.3529	0.2062	0.049*	
C2	−0.0721 (3)	0.0186 (2)	0.25006 (16)	0.0456 (6)	
H2	−0.1427	−0.0367	0.2527	0.055*	
C18	−0.1036 (3)	0.4969 (2)	0.21361 (15)	0.0417 (5)	
H18A	−0.1803	0.4503	0.2448	0.050*	
H18B	−0.0560	0.4440	0.1703	0.050*	
C24	−0.1388 (3)	0.7203 (2)	0.10202 (14)	0.0420 (5)	
H24	−0.0406	0.6732	0.0784	0.050*	
C12	0.2948 (3)	0.4785 (3)	0.02883 (14)	0.0437 (6)	
H12	0.2930	0.3922	0.0159	0.052*	
C3	0.0349 (3)	0.0461 (2)	0.18578 (15)	0.0415 (5)	
H3	0.0504	0.0128	0.1368	0.050*	
C22	−0.3700 (3)	0.9295 (2)	0.09812 (16)	0.0488 (6)	
H22	−0.4285	1.0230	0.0725	0.059*	
C11	0.2411 (3)	0.6078 (3)	−0.02778 (14)	0.0480 (6)	
H11	0.2027	0.6076	−0.0781	0.058*	
C8	0.3535 (3)	0.6060 (2)	0.12081 (14)	0.0411 (5)	
H8	0.3911	0.6069	0.1712	0.049*	
C26	0.5335 (3)	0.0771 (3)	0.34638 (16)	0.0516 (6)	
H26A	0.6071	0.0310	0.3911	0.077*	
H26B	0.5518	0.1675	0.3196	0.077*	
H26C	0.5518	0.0147	0.3076	0.077*	
C10	0.2440 (3)	0.7364 (3)	−0.01045 (15)	0.0481 (6)	
H10	0.2077	0.8232	−0.0486	0.058*	
C28	−0.1310 (3)	0.3356 (3)	0.43669 (17)	0.0521 (6)	
H28A	−0.1740	0.2694	0.4767	0.078*	
H28B	−0.1723	0.3451	0.3835	0.078*	
H28C	−0.1637	0.4289	0.4517	0.078*	
C21	−0.4296 (3)	0.8609 (3)	0.16978 (16)	0.0494 (6)	
H21	−0.5286	0.9081	0.1927	0.059*	
C23	−0.2250 (3)	0.8598 (3)	0.06492 (15)	0.0507 (6)	
H23	−0.1838	0.9065	0.0169	0.061*	
C9	0.3010 (3)	0.7351 (3)	0.06378 (16)	0.0494 (6)	
H9	0.3045	0.8214	0.0760	0.059*	
C27	0.1122 (3)	0.2549 (3)	0.51480 (15)	0.0514 (6)	
H27A	0.0806	0.3447	0.5339	0.077*	
H27B	0.2289	0.2185	0.5116	0.077*	
H27C	0.0705	0.1850	0.5525	0.077*	
C25	0.3401 (3)	−0.0277 (2)	0.42785 (17)	0.0523 (6)	
H25A	0.3585	−0.0990	0.3944	0.078*	
H25B	0.2302	−0.0066	0.4494	0.078*	
H25C	0.4146	−0.0648	0.4725	0.078*	

### Atomic displacement parameters (Å^2^)

**Table:**

	*U*^11^	*U*^22^	*U*^33^	*U*^12^	*U*^13^	*U*^23^
Ti	0.0237 (2)	0.0252 (2)	0.0335 (2)	−0.00713 (15)	−0.00057 (14)	−0.00083 (15)
N1	0.0292 (9)	0.0253 (8)	0.0399 (10)	−0.0091 (7)	0.0005 (7)	−0.0025 (7)
N2	0.0262 (9)	0.0274 (8)	0.0372 (9)	−0.0070 (7)	0.0035 (7)	0.0006 (7)
N4	0.0239 (9)	0.0276 (8)	0.0377 (9)	−0.0066 (7)	−0.0042 (7)	−0.0027 (7)
N3	0.0256 (9)	0.0301 (8)	0.0362 (9)	−0.0094 (7)	−0.0019 (7)	−0.0034 (7)
N5	0.0333 (10)	0.0314 (9)	0.0390 (10)	−0.0051 (7)	−0.0053 (8)	−0.0041 (7)
N6	0.0346 (10)	0.0323 (9)	0.0417 (10)	−0.0106 (8)	0.0048 (8)	−0.0036 (8)
C7	0.0294 (11)	0.0392 (11)	0.0359 (11)	−0.0150 (9)	0.0092 (9)	−0.0055 (9)
C14	0.0357 (13)	0.0491 (13)	0.0412 (12)	−0.0212 (10)	−0.0009 (10)	−0.0135 (10)
C17	0.0295 (11)	0.0238 (9)	0.0388 (11)	−0.0038 (8)	−0.0022 (9)	−0.0063 (8)
C16	0.0284 (11)	0.0293 (10)	0.0295 (10)	−0.0081 (8)	0.0014 (8)	−0.0055 (8)
C19	0.0280 (11)	0.0297 (10)	0.0397 (11)	−0.0088 (9)	−0.0087 (9)	−0.0048 (9)
C4	0.0310 (11)	0.0231 (9)	0.0406 (11)	−0.0034 (8)	−0.0017 (9)	−0.0041 (8)
C5	0.0320 (12)	0.0293 (10)	0.0346 (11)	−0.0031 (9)	0.0037 (9)	−0.0034 (9)
C13	0.0282 (11)	0.0407 (11)	0.0381 (11)	−0.0107 (9)	−0.0051 (9)	−0.0025 (9)
C1	0.0340 (12)	0.0320 (11)	0.0532 (13)	−0.0132 (9)	0.0050 (10)	−0.0044 (10)
C20	0.0345 (13)	0.0414 (12)	0.0406 (12)	−0.0122 (10)	0.0007 (9)	−0.0024 (10)
C15	0.0390 (13)	0.0340 (11)	0.0376 (11)	−0.0135 (9)	0.0010 (9)	−0.0099 (9)
C6	0.0291 (12)	0.0436 (12)	0.0463 (13)	−0.0133 (10)	0.0064 (10)	−0.0011 (10)
C2	0.0352 (13)	0.0329 (11)	0.0731 (17)	−0.0155 (10)	0.0012 (11)	−0.0132 (11)
C18	0.0368 (13)	0.0297 (11)	0.0583 (14)	−0.0097 (9)	−0.0198 (11)	−0.0014 (10)
C24	0.0363 (13)	0.0433 (12)	0.0456 (13)	−0.0113 (10)	0.0054 (10)	−0.0106 (10)
C12	0.0536 (15)	0.0445 (12)	0.0419 (13)	−0.0275 (11)	0.0040 (11)	−0.0110 (10)
C3	0.0397 (13)	0.0320 (11)	0.0529 (14)	−0.0067 (10)	−0.0047 (10)	−0.0135 (10)
C22	0.0508 (16)	0.0305 (11)	0.0593 (15)	−0.0058 (11)	−0.0192 (12)	0.0007 (11)
C11	0.0499 (15)	0.0611 (15)	0.0364 (12)	−0.0279 (12)	−0.0007 (10)	−0.0011 (11)
C8	0.0461 (14)	0.0471 (13)	0.0350 (12)	−0.0212 (11)	0.0086 (10)	−0.0117 (10)
C26	0.0335 (13)	0.0563 (15)	0.0520 (15)	0.0058 (11)	−0.0068 (11)	−0.0097 (12)
C10	0.0436 (14)	0.0430 (13)	0.0499 (14)	−0.0144 (11)	0.0071 (11)	0.0052 (11)
C28	0.0389 (14)	0.0562 (15)	0.0612 (16)	−0.0160 (12)	0.0146 (12)	−0.0160 (12)
C21	0.0310 (13)	0.0436 (13)	0.0650 (16)	0.0033 (10)	−0.0035 (11)	−0.0137 (12)
C23	0.0675 (18)	0.0457 (13)	0.0394 (13)	−0.0263 (13)	−0.0039 (12)	0.0049 (11)
C9	0.0580 (16)	0.0380 (12)	0.0550 (15)	−0.0216 (11)	0.0110 (12)	−0.0101 (11)
C27	0.0571 (17)	0.0521 (14)	0.0419 (13)	−0.0155 (12)	0.0052 (12)	−0.0072 (11)
C25	0.0592 (17)	0.0276 (11)	0.0680 (17)	−0.0105 (11)	−0.0166 (13)	−0.0034 (11)

### Geometric parameters (Å, º)

**Table:**

Ti—N6	1.8987 (18)	C20—H20	0.9300
Ti—N5	1.9091 (19)	C15—H15	0.9300
Ti—N3	2.1011 (17)	C6—H6A	0.9700
Ti—N1	2.1134 (19)	C6—H6B	0.9700
Ti—N4	2.2449 (19)	C2—C3	1.386 (3)
Ti—N2	2.2897 (18)	C2—H2	0.9300
N1—C1	1.350 (3)	C18—H18A	0.9700
N1—C4	1.379 (3)	C18—H18B	0.9700
N2—C5	1.277 (3)	C24—C23	1.380 (3)
N2—C6	1.482 (3)	C24—H24	0.9300
N4—C17	1.282 (3)	C12—C11	1.384 (3)
N4—C18	1.479 (3)	C12—H12	0.9300
N3—C13	1.351 (3)	C3—H3	0.9300
N3—C16	1.387 (3)	C22—C23	1.361 (4)
N5—C25	1.455 (3)	C22—C21	1.377 (4)
N5—C26	1.456 (3)	C22—H22	0.9300
N6—C27	1.451 (3)	C11—C10	1.373 (4)
N6—C28	1.459 (3)	C11—H11	0.9300
C7—C12	1.379 (3)	C8—C9	1.385 (3)
C7—C8	1.382 (3)	C8—H8	0.9300
C7—C6	1.508 (3)	C26—H26A	0.9600
C14—C13	1.389 (3)	C26—H26B	0.9600
C14—C15	1.399 (3)	C26—H26C	0.9600
C14—H14	0.9300	C10—C9	1.369 (4)
C17—C16	1.419 (3)	C10—H10	0.9300
C17—H17	0.9300	C28—H28A	0.9600
C16—C15	1.385 (3)	C28—H28B	0.9600
C19—C20	1.375 (3)	C28—H28C	0.9600
C19—C24	1.384 (3)	C21—H21	0.9300
C19—C18	1.504 (3)	C23—H23	0.9300
C4—C3	1.383 (3)	C9—H9	0.9300
C4—C5	1.432 (3)	C27—H27A	0.9600
C5—H5	0.9300	C27—H27B	0.9600
C13—H13	0.9300	C27—H27C	0.9600
C1—C2	1.381 (3)	C25—H25A	0.9600
C1—H1	0.9300	C25—H25B	0.9600
C20—C21	1.382 (3)	C25—H25C	0.9600
			
N6—Ti—N5	101.90 (8)	C7—C6—H6A	108.7
N6—Ti—N3	97.59 (8)	N2—C6—H6B	108.7
N5—Ti—N3	95.05 (7)	C7—C6—H6B	108.7
N6—Ti—N1	94.23 (8)	H6A—C6—H6B	107.6
N5—Ti—N1	97.39 (8)	C1—C2—C3	106.5 (2)
N3—Ti—N1	160.70 (7)	C1—C2—H2	126.8
N6—Ti—N4	92.44 (7)	C3—C2—H2	126.8
N5—Ti—N4	163.77 (7)	N4—C18—C19	116.14 (17)
N3—Ti—N4	75.34 (7)	N4—C18—H18A	108.3
N1—Ti—N4	89.00 (7)	C19—C18—H18A	108.3
N6—Ti—N2	165.25 (8)	N4—C18—H18B	108.3
N5—Ti—N2	89.27 (8)	C19—C18—H18B	108.3
N3—Ti—N2	90.90 (7)	H18A—C18—H18B	107.4
N1—Ti—N2	74.60 (7)	C23—C24—C19	120.7 (2)
N4—Ti—N2	78.01 (7)	C23—C24—H24	119.6
C1—N1—C4	105.19 (18)	C19—C24—H24	119.6
C1—N1—Ti	137.79 (16)	C7—C12—C11	120.8 (2)
C4—N1—Ti	116.42 (13)	C7—C12—H12	119.6
C5—N2—C6	117.90 (18)	C11—C12—H12	119.6
C5—N2—Ti	112.70 (13)	C4—C3—C2	106.4 (2)
C6—N2—Ti	129.23 (15)	C4—C3—H3	126.8
C17—N4—C18	121.51 (17)	C2—C3—H3	126.8
C17—N4—Ti	113.45 (14)	C23—C22—C21	119.7 (2)
C18—N4—Ti	124.76 (13)	C23—C22—H22	120.2
C13—N3—C16	105.68 (17)	C21—C22—H22	120.2
C13—N3—Ti	138.25 (14)	C10—C11—C12	120.7 (2)
C16—N3—Ti	115.35 (13)	C10—C11—H11	119.7
C25—N5—C26	110.80 (19)	C12—C11—H11	119.7
C25—N5—Ti	120.62 (16)	C7—C8—C9	121.1 (2)
C26—N5—Ti	126.40 (15)	C7—C8—H8	119.4
C27—N6—C28	110.83 (19)	C9—C8—H8	119.4
C27—N6—Ti	120.95 (16)	N5—C26—H26A	109.5
C28—N6—Ti	127.38 (16)	N5—C26—H26B	109.5
C12—C7—C8	117.9 (2)	H26A—C26—H26B	109.5
C12—C7—C6	121.7 (2)	N5—C26—H26C	109.5
C8—C7—C6	120.3 (2)	H26A—C26—H26C	109.5
C13—C14—C15	106.6 (2)	H26B—C26—H26C	109.5
C13—C14—H14	126.7	C9—C10—C11	119.1 (2)
C15—C14—H14	126.7	C9—C10—H10	120.5
N4—C17—C16	118.74 (18)	C11—C10—H10	120.5
N4—C17—H17	120.6	N6—C28—H28A	109.5
C16—C17—H17	120.6	N6—C28—H28B	109.5
C15—C16—N3	110.44 (18)	H28A—C28—H28B	109.5
C15—C16—C17	133.02 (19)	N6—C28—H28C	109.5
N3—C16—C17	116.54 (17)	H28A—C28—H28C	109.5
C20—C19—C24	118.36 (19)	H28B—C28—H28C	109.5
C20—C19—C18	121.14 (19)	C22—C21—C20	120.0 (2)
C24—C19—C18	120.5 (2)	C22—C21—H21	120.0
N1—C4—C3	110.47 (19)	C20—C21—H21	120.0
N1—C4—C5	116.31 (19)	C22—C23—C24	120.4 (2)
C3—C4—C5	133.2 (2)	C22—C23—H23	119.8
N2—C5—C4	119.35 (19)	C24—C23—H23	119.8
N2—C5—H5	120.3	C10—C9—C8	120.4 (2)
C4—C5—H5	120.3	C10—C9—H9	119.8
N3—C13—C14	111.09 (19)	C8—C9—H9	119.8
N3—C13—H13	124.5	N6—C27—H27A	109.5
C14—C13—H13	124.5	N6—C27—H27B	109.5
N1—C1—C2	111.5 (2)	H27A—C27—H27B	109.5
N1—C1—H1	124.3	N6—C27—H27C	109.5
C2—C1—H1	124.3	H27A—C27—H27C	109.5
C19—C20—C21	120.8 (2)	H27B—C27—H27C	109.5
C19—C20—H20	119.6	N5—C25—H25A	109.5
C21—C20—H20	119.6	N5—C25—H25B	109.5
C16—C15—C14	106.20 (18)	H25A—C25—H25B	109.5
C16—C15—H15	126.9	N5—C25—H25C	109.5
C14—C15—H15	126.9	H25A—C25—H25C	109.5
N2—C6—C7	114.20 (18)	H25B—C25—H25C	109.5
N2—C6—H6A	108.7		
